# A student-initiated objective structured clinical examination as a sustainable cost-effective learning experience

**DOI:** 10.1080/10872981.2018.1440111

**Published:** 2018-02-26

**Authors:** Claire B. Lee, Lorenzo Madrazo, Usman Khan, Tharshika Thangarasa, Meghan McConnell, Karima Khamisa

**Affiliations:** ^a^ Department of Medicine, McGill University, Montreal, QC, Canada; ^b^ Faculty of Medicine, University of Ottawa, Ottawa, ON, Canada

**Keywords:** OSCE, undergraduate medical education, learning, peer-assessment, clinical skills

## Abstract

**Background**: The objective structured clinical examination (OSCE) has gained widespread use as a form of performance assessment. However, opportunities for students to participate in practice OSCEs are limited by the financial, faculty and administrative investments required.

**Objectives**: To determine the feasibility and acceptability of a student-run mock OSCE (MOSCE) as a learning experience for medical students of all 4 years.

**Design**: We conducted a five-station MOSCE for third-year students. This involved fourth-year students as examiners and first-/second-year students as standardized patients (SPs). Each examiner scored examinees using a checklist and global rating scale while providing written and verbal feedback. MOSCE stations and checklists were designed by students and reviewed by a faculty supervisor. Following the MOSCE, participants completed surveys which elucidated their perceptions on the roles they took during the MOSCE.

**Results**: Fifty examinees participated in the MOSCE. Of these, 42 (84%) consented to participate in the study and submitted completed questionnaires. Twenty-four examiners participated in the OSCE and consented to participate in the study, with 22 (92%) submitting completed questionnaires. Fifty-three of 60 SPs (88%) agreed to take part in this study, and 51 (85%) completed questionnaires. The internal consistency of the five-station OSCE was calculated as a Cronbach’s alpha of 0.443. Students commented positively on having the opportunity to network and engage in mentorship activities and reinforce clinical concepts.

**Conclusions**: Examinees, examiners, and SPs all perceived the MOSCE to be a beneficial learning experience. We found the MOSCE to be a feasible and acceptable means of providing additional OSCE practice to students prior to higher-stakes evaluations.

## Introduction

The objective structured clinical examination (OSCE) is accepted as a robust method of performance assessment in medical education [1]. Developed in 1975 by Harden and his colleagues [2], the OSCE has since been proven to serve as a reliable, valid, and accurate measure of clinical skills [3–5], leading to its use for summative and formative purposes.

While OSCEs serve as a powerful learning opportunity for medical trainees, there are a variety of challenges with implementing OSCEs regularly within medical curricula. For example, there are significant financial costs associated with implementing an OSCE [3]. The majority of expenses often result from compensating standardized patients (SPs), examiners, support staff, and the OSCE lead [6]. Furthermore, even in formative, low-stakes settings, OSCEs can be anxiety-inducing experiences for students [7,8]. In light of these factors, we sought to implement a mock OSCE (MOSCE) wherein the organizing team and all the participants – examiners, examinees, and SPs – consist of students.

The concept of using students as either examiners or as SPs is not a novel one. A recent scoping review on peer-assessment in OSCEs found that using peer evaluators in an OSCE is appropriate in formative settings, promoting learning to all the students involved [9]. Though the literature on student SPs is less extensive, medical students have also been shown to be reliable and cost-effective SPs in formative OSCE settings [10]; furthermore, as with student examiners, Mavis and colleagues [11] reported that student SPs benefited from learning experiences gained during the OSCE encounter.

At our institution, we aimed to develop a sustainable learning intervention to increase opportunities for OSCE practice. Our MOSCE is designed and implemented by students, synthesizing the concepts of students as peer evaluators and as SPs. The primary objective of the present study was to evaluate the feasibility and acceptability of our student-led MOSCE as an educational event for students in all 4 years of the undergraduate medical program.

## Methods

### Study context and participants

At the University of Ottawa, the undergraduate medical program is of a 4-year duration wherein the first two years, pre-clerkship, are spent on classroom learning and the last two years, clerkship, are spent gaining practical experience in the clinical setting. The Faculty of Medicine currently administers formative OSCEs in Years I, II, and III of the training program and summative OSCEs in Years II and IV. The Year III OSCE introduces clerkship students to management and counselling stations, which are markedly more complex station types than those experienced in pre-clerkship. While formative, the Year III OSCE serves to identify at risk students and thus, borderline or failing performance in this OSCE may result in counselling, closer scrutiny, extra coaching, and/or remediation. However, prior to the Year III OSCE there are no opportunities for students to practice the new complex station types. We identified this gap as a suitable niche for our MOSCE.

University of Ottawa third-year medical students were recruited as examinees to gain exposure to clerkship-level stations prior to their Year III OSCE. Fourth-year volunteers were recruited to act as examiners. First- and second-year volunteers were recruited to be SPs in the MOSCE, due to their medical knowledge and their interest in observing a clerkship-level OSCE. The authors were excluded from participating in the MOSCE.

**Figure 1.**
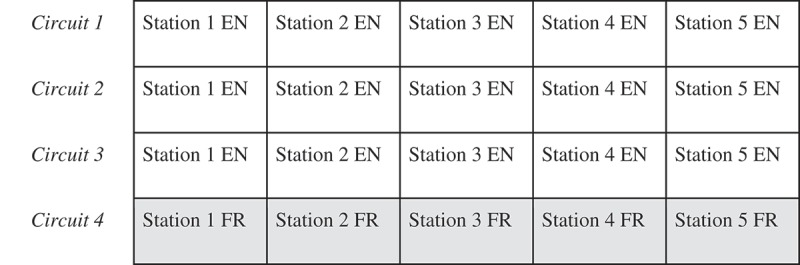
OSCE circuit schematic. This figure illustrates how a single iteration of the OSCE was structured. During each iteration, four identical circuits with five stations each were run simultaneously as shown (three were done in English, while the fourth was done in French). This allowed for 20 examinees to participate in each iteration. Three identical iterations were carried out sequentially, allowing a maximum of 60 student examinees to participate over the course of the evening.

Students in each of the above groups were signed up on a first-come-first-serve basis and received training as described further below. The same examiners remained throughout all three iterations of the faculty-led OSCE (Figure 1). The examinees and SPs each took part in only one iteration.

Study participants were recruited on a voluntary basis from the students taking part in the MOSCE. The study was approved by the Ottawa Health Science Network Research Ethics Board. All study participants provided informed consent. Participant identification numbers were assigned to anonymize data. Data collected from non-consenting students were discarded and not included in analysis.

One of the goals of the student-led MOSCE was to prepare third-year students for the upcoming formative Year III OSCE. Since the year III OSCE at our institution is modelled after the OSCE portion of the Medical Council of Canada Qualifying Examination (MCCQE), we also selected station content to represent the major specialties tested on the MCCQE. Specific topics were chosen from these specialties such that the 5 stations combined would allow for a mix of history-taking, physical examination, counselling, and management stations. Stations for the 2017 MOSCE were as follows: (1) Paediatric cystic fibrosis history; (2) General Surgery cholecystitis management; (3) Family Medicine lower back pain physical exam; (4) Psychiatry depression counselling; and (5) Internal/Emergency Medicine ST elevation myocardial infarction (STEMI) management.

Scoresheets for each station began with a stem containing a description of the clinical scenario and instructions for the examinee. This was followed by a checklist and then a global rating scale (GRS). The GRS used a six-point scale ranging from 1 = inferior to 6 = excellent. Checklists were each conceptualized and written by individual Year II and IV medical students (first to fourth authors), modified by a Year IV medical student (first author), and finally further reviewed and approved by a faculty advisor (last author), an experienced OSCE chief examiner for undergraduate medical education. The review process took seven hours of faculty time.

Figure 1 indicates the organization of the MOSCE. Each station totalled 10 min in length, broken down as follows: 1 min for reading the scenario, 7 min for completing the encounter, and 2 min for receiving feedback from the examiner. Thus, each iteration lasted 50 min. The entire event was coordinated by students without the need for further administrative support.

A few weeks in advance of the MOSCE, SPs and examiners were assigned to specific stations and were sent their cases with detailed instructions. Examinees received information orienting them to the MOSCE schedule and the breadth of specialties and station types they may encounter, but the specific diagnoses of the stations were not divulged. Each group of students received training/briefing on the night of the MOSCE prior to participating in the event. Examiners attended a 30-minute didactic session given by a faculty advisor (last author) that focused on techniques for assessing examinees and giving verbal and written feedback. Examinees were briefly reminded of the station timings and instructed on how to navigate their MOSCE circuits. SPs were asked to only reveal history items or physical examination findings if specifically elicited by the examinee. Examiners and SPs were offered the opportunity to ask questions specific to their stations. After finishing their respective MOSCE activities, each group of study participants had the opportunity to complete surveys regarding their experience as examiners, examinees, and SPs. At this time, examinees received a copy of their station scoresheets as written feedback. All research materials were handled by hired research assistants such that the authors were unable to link research materials to individual study participants.

### Measures

Questionnaires were comprised of 11 close-ended questions for examiners, 12 for examinees, and 9 for SPs. Close-ended questions consisted of statements with response options arranged in a five-point Likert scale ranging from ‘Strongly Disagree’ (1) to ‘Strongly Agree’ (5). In the examinee questionnaire, close-ended questions were adapted from Moineau and colleagues [12] (see Table 1). For examiner and SP questionnaires, close-ended questions were based on questionnaires designed by Burgess and colleagues [13] (see Tables 2 and 3, respectively). In addition, each questionnaire contained three open-ended questions that elicited perceptions on the involvement of all 4 years of medical students, ways to improve the MOSCE, and on the MOSCE in general.

**Table 1.** Student examinee perceptions.

Item	Min	Max	SD	Mean*
Comfortable being evaluated on my history-taking skills	3	5	0.64	4.37
Comfortable being evaluated on communication skills	3	5	0.65	4.43
Comfortable being evaluated on physical examination skills	3	5	0.64	4.37
Peer OSCE acceptable for formative purposes	1	5	1.15	4.24
Peer OSCE would be acceptable for summative purposes	1	5	1.41	2.54
Received constructive feedback	3	5	0.68	4.57
Feedback given in appropriate manner	2	5	0.58	4.70
Prefer faculty examiner instead of senior medical student	1	5	1.26	3.20
Tension with examiner that would be detrimental to feedback or evaluation	1	5	0.92	1.59
Peer evaluation OSCEs are worthwhile	3	5	0.68	4.46
Spent time preparing for this mock OSCE	1	5	1.12	1.70
Instructions provided were clear and prepared me adequately	2	5	0.92	4.02

*Mean scores on a 5-point scale (1 = strongly disagree, 5 = strongly agree). SD: standard deviation.

**Table 2.** Student examiner perceptions.

Item	Min	Max	SD	Mean*
Apply prior knowledge	4	5	0.47	4.68
Build on prior knowledge	3	5	0.73	4.09
Challenged my prior knowledge	1	5	0.96	3.27
Developed my clinical skills	1	5	1.00	3.77
Developed confidence in clinical skills	2	5	0.92	4.31
Helpful in preparing me for my future examinations	3	5	0.58	4.55
Interesting learning activity	4	5	0.34	4.86
I was active in participating during the OSCE	3	5	0.64	4.70
Adequate training and preparation materials	3	5	0.55	4.68
Confident to make a judgment on student performance	3	5	0.66	4.5
Confident to provide feedback	1	5	0.65	4.59

*Mean scores on a 5-point scale (1 = strongly disagree, 5 = strongly agree). SD: standard deviation.

**Table 3.** Student standardized patient perceptions.

Item	Min	Max	SD	Mean*
Allowed me to apply prior knowledge	1	5	0.88	4.27
Allowed me to build on my prior knowledge	3	5	0.60	4.55
Challenged my prior knowledge	1	5	1.01	3.90
Developed my clinical skills	2	5	0.83	4.33
Developed my confidence in clinical skills	1	5	0.94	3.98
Helpful in preparing me for my future examinations	2	5	0.65	4.63
Interesting learning activity	4	5	0.32	4.88
Adequate training and preparation materials	3	5	0.64	4.47
Active in participating during the OSCE	3	5	0.67	4.55

*Mean scores on a 5-point scale (1 = strongly disagree, 5 = strongly agree). SD: standard deviation.

Examinees were asked to provide a self-assessment score by rating their performance on a six-point Likert Scale ranging from ‘Inferior’ (1) to ‘Excellent’ (6), corresponding to the examiner GRS. Completed checklist items were summed and expressed as a percentage of total checklist items as an objective indicator of performance to be compared with the GRS score awarded by the examiner. However, these data will not be discussed in this report.

### Data analysis

Mean scores and percentages of responses to close-ended questions on questionnaires were calculated. The responses to open-ended questionnaire items were coded and analysed thematically by two authors and any discrepant items were discussed and resolved by consensus with two other authors. Cronbach’s α was measured to ensure there was some consistency within the construct of the student-developed MOSCE. The α for the MOSCE as a whole was calculated using the mean proportion checklist completion scores of each of the five stations. Statistical analyses were conducted using SPSS version 24.

## Results

### Participants

A total of 50 examinees participated in the MOSCE. Of those who participated, 47 (94%) consented to be a part of this study and 42 (84%) submitted completed questionnaires. Twenty-four examiners participated in the MOSCE and all consented to take part in the research component, with 22 (92%) submitting completed questionnaires. Further, there were a total of 60 SPs, 53 (88%) of whom agreed to take part in this study, and 51 (85%) of whom completed questionnaires.

### Reliability analysis

The internal consistency of the five station MOSCE as a whole was calculated as a Cronbach’s alpha of 0.443 based on the mean proportion checklist completion scores of each station. This value is interpreted as having low reliability [14].

### Responses to close-ended questions

#### Examinees

Most examinees found the MOSCE to be a positive experience and learning environment, as shown in Table 1. They felt comfortable being evaluated by their peers for formative purposes (M = 4.24, SD = 1.15) but did not feel it would be acceptable for summative purposes (M = 2.54, SD = 1.41). Examinees perceived that their examiners provided constructive (M = 4.57, SD = 0.68) and appropriate feedback (M = 4.70, SD = 0.58). Many did not prepare for the MOSCE (M = 1.70, SD = 1.12) but still felt there was value in peer-evaluated OSCEs (M = 4.46, SD = 0.68). Most students disagreed that there was tension between themselves and their examiners that would affect their evaluation (M = 1.59, SD = 0.92).

#### Examiners

Mean scores (M), standard deviations (SD), and maximum and minimum values for examiner responses to each questionnaire item are summarized in Table 2. Examiners agreed that participating in the MOSCE allowed them to apply (M = 4.68, SD = 0.47) and build (M = 4.09, SD = 0.73) on prior knowledge. However, they only moderately agreed that it challenged their prior knowledge (M = 3.27, SD = 0.96) and developed their clinical skills (M = 3.77, SD = 1.00). Examiners perceived the MOSCE as a helpful learning activity for future examinations (M = 4.55, SD = 0.58). They also felt adequately prepared (M = 4.68, SD = 0.55) and sufficiently confident to assess student performance (M = 4.50, SD = 0.66) and provide feedback (M = 4.59, SD = 0.65).

#### Standardized patients

The first- and second-year medical students who served as SPs also perceived several benefits from participating in the MOSCE (Table 3). SPs agreed that the MOSCE allowed them to apply (M = 4.27, SD = 0.88) and build upon their prior knowledge (M = 4.55, SD = 0.60). They perceived the MOSCE to be helpful in the development of their clinical skills (M = 4.33, SD = 0.83) and in preparing them for future examinations (M = 4.63, SD = 0.65). They also felt adequately trained in their role as SPs (M = 4.47, SD = 0.64).

### Responses to open-ended questions

Overall, the MOSCE was very well received by the student body. The feedback collected following the event indicated benefits for all the groups of participants involved: fourth-year examiners, third-year examinees, and first- and second-year SPs. These benefits could be grouped into several general categories: teaching, learning, and collegiality. Feedback also highlighted some concerns regarding OSCE characteristics, training, and awkward interactions (Table 4).

**Table 4.** Summary of major themes that emerged from student comments with illustrative quotes.

**Theme**	**Representative Quotes**
**Benefits**	
**Teaching**	“I thought it was good, prepares pre-clerks for their OSCE, helps [fourth year students] becoming teachers. And obviously [third year students] for OSCE.” - 4^th^ year examiner
	“I really value being part of teaching. Helps enforce concepts and teaches me a lot as well too.” - 4th year examiner
**Learning**	“It was a great experience being a fourth year as an examiner [and] being able to apply clinical knowledge/skills in this setting. It was most beneficial to third years. First year students thought it was a good experience for them to get a feeling of how the whole OSCE works.” - 4th year examiner
	“It was a great experience. It was very helpful especially the management stations which I had no experience with in preclerkship. I would rather struggle with it now than on an OSCE for marks.” - 3rd year examinee
	“I thought it was a great experience. I was able to talk to the 4th year examiner who was incredibly helpful and knowledgeable. It was a great learning opportunity from that perspective as well.” - 1st/2nd year SP
**Collegiality**	“It was great to meet some 1st and 2nd years as SPs and to see how the 3rd years are progressing.” - 4th year examiner
	“I thought that having peers for my first 3rd year practise OSCE was a low stress environment. I would have been much more nervous with an official examiner. However, this would not have been ideal for a summative OSCE.” - 3rd year examinee
	“Good experience to meet other students! And learn from 4th years.” - 1st/2nd year SP
	“It was a pleasure to be involved in something that benefits the entire Faculty of Medicine.” - 4th year examiner
	“[I] really like that all years come together to help each other. ” - 1st/2nd year SP
**Areas of Concern**	
**OSCE Characteristics**	“I find that I could have given more feedback if we had more time.” - 4th year examiner
	“[Would prefer] more time for feedback at the end - helpful but found time short for this.” - 3rd year examinee
	“Possibly [have] more stations, great practice.” - 3rd year examinee “My scoring checklist was lacking in several areas.” - 4th year examiner
**Training**	“More teaching on global assessment for borderline satisfactory vs borderline unsatisfactory.” - 4th year examiner
	“I think that there needs to be more of an orientation to how a 3rd year OSCE works in terms of management stations and how to manage time effectively for a 7 minute station.” - 3rd year examinee
	“More training to SPs regarding how much information to give to the patient (eg SP disclosed symptom of wheezing when student did not ask).” - 4th year examiner “Brief SPs on their specific station as some people may not know how to act out certain physical findings (ex. Murphy’s sign).” - 4th year examiner
**Awkward Interaction**	“It made it a little awkward, especially with those I’m friends with, but still worthwhile and beneficial.” - 3rd year examinee“It was awkward when I knew one of the examiners personally.” - 3rd year examinee

#### Teaching

While all examiners surveyed provided positive remarks (e.g., ‘great experience,’ ‘It was a pleasure to participate’), three examiners specifically commented on the valuable teaching opportunity they had while participating in the MOSCE. Four examiners commented on how being an examiner helped reinforce concepts and reflect on how much they have developed.

#### Learning

Notably, one examiner commented on how the students involved ‘all learned something appropriate to their level.’ This was echoed by 12 SPs who specified how they enjoyed learning more about OSCEs from interacting with their third- and fourth-year colleagues. Most importantly, examinees appreciated being exposed to novel station types (i.e., management stations) in a low-stakes setting, saying that they’d rather ‘struggle with it now than on an OSCE for marks.’ They also appreciated the feedback that their fourth-year colleagues provided them. The immediate return of scoresheets after completion of all five stations was well received.

#### Collegiality

Examiners, examinees, and SPs appreciated the opportunity to network and collaborate with students in all 4 years of the programme. Examiners and SPs valued the collegiality they experienced when ‘all years come together to help each other.’ As one examiner stated, ‘It was a pleasure to be involved in something that benefits the entire Faculty of Medicine.’

#### OSCE characteristics

General feedback regarding OSCE characteristics consisted of examinees and examiners commenting that they would have liked more time for feedback. Examinees also requested more stations in the MOSCE for practice.

#### Training

An examiner recommended improved training on the scoring system, specifically suggesting ‘more teaching on [the] global assessment for borderline satisfactory vs. borderline unsatisfactory.’ Examinees and examiners noted that some examinees were unsure of how to approach management stations, and/or how to use the standardized nurse present in one of these stations. Another suggestion by three SPs and two examiners was to provide better training to SPs prior to the MOSCE, given that some of the students seemed to be unfamiliar with how to act out certain physical findings and how much information to reveal during history taking stations.

#### Awkward interactions

Whereas examinees generally disagreed that there were tensions between them and student examiners, two examinees reported that they experienced an awkward interaction when their examiner was someone they were familiar with (it was awkward when I knew one of the examiners personally).

## Discussion

The objective of our study was to assess the feasibility and acceptability of our student-initiated peer-assisted MOSCE. Our quantitative and qualitative data suggest that both examinees and non-examinees perceived several benefits from participating in the MOSCE. First- and second-year students observed examinees while reflecting on their own clinical skills, whereas fourth-year students gained important experience in a teaching role before their transition into residency.

All three groups of students noted how the MOSCE was a unique evening for networking and collaboration between students from every year in the undergraduate medical programme. First- and second-year students appreciated networking with fourth-year students, who are nearing the end of the program and have many insights to share. We believe that the strong collegiality observed was a major contributor to the acceptability of the MOSCE.

From a financial perspective, the MOSCE proved to be more cost effective compared to a traditional faculty-led OSCE, the latter costing around $96,000 CAD ($75,000 USD) for a class size of 166 students at our institution, or $578 CAD ($450 USD) per examinee [Personal Communication, Ottawa Examination Centre]). In contrast, the costs of organizing and implementing this MOSCE with the capacity to accommodate 60 examinees totalled roughly $600 CAD ($470 USD), or $10 CAD ($8 USD) per examinee, the vast majority of which was spent on providing dinner to participants. As resource stewardship becomes increasingly critical to healthcare and healthcare education, our MOSCE serves as an example of a creative strategy to meet a learning need in a cost-effective way.

While we recognize the benefits of this MOSCE, it is also important to address concerns raised by students regarding training, OSCE characteristics, and awkward interactions. In terms of training, there were comments pertaining to each of the three roles in the MOSCE. First, examiners expressed interest in learning how to better score borderline candidates. Although the MOSCE was completely formative in nature, enhanced examiner training would likely be beneficial in the future, both for examinees seeking quantitative and qualitative feedback and for examiners developing their skillset as teachers. Second, examinees reported being unaware of what a management station entailed. Exposure to this type of station is quite limited in Year I and II OSCEs; however, the Faculty of Medicine may be persuaded to offer further support of a dedicated MOSCE in the future devoted to strictly management scenarios. Third, examiners noted that some SPs were unfamiliar with how much history to divulge or how to demonstrate physical exam findings. This suggests that sending written instructions and providing a brief orientation were insufficient in preparing first- and second-year students to be SPs in the MOSCE; implementing more formalized training similar to the 30-minute model used by Mavis, Ogle, Lovell, and Madden [10] may improve the consistency in SP performance in future iterations.

With regards to OSCE characteristics, students in each of the three roles suggested that there was an inadequate amount of time for verbal feedback post-encounter, although the time allocated reflected that of the faculty-led OSCEs. There were also requests for a greater number of stations. These findings reflected the keen interest students had in getting more practice and receiving/giving more feedback in the context of this student-led initiative. It is also possible that fourth-year students were less efficient at giving verbal feedback than experienced faculty, and it may be worthwhile to train them in focusing on key points to communicate to examinees in a limited timeframe. With regards to the MOSCE cases themselves, there was one comment from an examiner who felt that the checklist for their station was lacking. While the student-designed checklists allow for the return of the scoresheets to examinees, they are likely of lower quality than cases that have been designed by experienced faculty, piloted, and subjected to rigorous statistical analyses. Hence, this reinforces that the use of this student-initiated peer-OSCE construct is most appropriate for low-stakes formative situations.

Finally, although most examinees disagreed that there was tension between themselves and their examiners, two students reported that being evaluated by near-peers with whom they had personal relationships made for an awkward interaction. Cushing and colleagues [15] have also described mixed findings in this regards; in their OSCE, some examinees felt that having their classmates be their peer assessors helped them relax whereas others perceived increased pressure. In a study by Moineau and colleagues [12], the authors administered a post-OSCE questionnaire to second-year examinees that was adapted for use in the present study; they found that most examinees did not perceive tension with their fourth-year student examiners, and furthermore there were no comments from examinees to suggest the contrary. It is possible that larger gaps in training level between examinees and examiners decrease the potential for awkward or tense interactions. Although we could entertain the possibility of recruiting residents instead of fourth-year students to act as examiners in our MOSCE, we believe that this would detract from the collegiality and unique learning opportunities inherent in this student-led initiative. All in all, the occasional awkward interactions inherent in this type of OSCE do not eclipse its benefits to students, but should serve to reinforce previous recommendations limiting this construct to a low-stakes, formative setting [9].

The Cronbach’s alpha calculated for the MOSCE as a whole was 0.443. An alpha of greater than 0.7–0.8 is considered to be necessary for a formal OSCE held for the purpose of evaluation [3]. To the best of our knowledge, no previous authors have reported measures of internal consistency for peer-assisted OSCEs. The purpose of calculating the alpha for this present study was to assess whether or not there was some degree of internal consistency in our construct, with the understanding that factors were present which would increase the variability in examinee performance from one station to the next (for example, each examinee was only halfway through a year’s worth of core clinical rotations, which they experienced in a different order than many of their peers). The small number of stations and the variety of competencies being assessed may have contributed to the lower alpha as well [16]. Since the goal of the MOSCE was not to evaluate or discriminate between high- versus low-performing students, achieving a high alpha was less of a focus in the present study.

Our study is not without limitations. It should be noted that the sample of students who participated did so voluntarily, hence their motivation to learn in this peer-assisted context may have contributed to the amount of positive feedback received. Furthermore, this group of students was from a single site, limiting generalizability. Finally, as data gathering was limited to the night of the MOSCE, it is unknown whether examinees still perceived the MOSCE as beneficial after undergoing the year 3 faculty-led OSCE.

This study has brought to light some unanswered questions to be addressed with future research. Although this OSCE is one of several that has used students as examiners, it is not known whether this early exposure to the assessor role prepares students to be assessors in OSCE or non-OSCE settings in their residency. Furthermore, it would be interesting to evaluate the efficacy of this MOSCE in improving the performance of both examinees and SPs in their future summative faculty-led OSCEs.

## Conclusions

This student-initiated MOSCE was highly accepted among fourth-year examiners, third-year examinees, and first- and second-year SPs, and served as a unique learning and mentorship opportunity for all students in the MD program at our institution. Its cost-effectiveness ensures that it will continue to represent a feasible means of preparing third-year students for higher-stakes clerkship OSCEs. Comments regarding the learning benefits perceived by SPs and examiners highlight the potential utility of these roles as opportunities for learning in an OSCE. Student participation in future iterations of this MOSCE could be correlated with performance in faculty-led OSCEs to better determine its efficacy as a preparatory activity.
